# Clinical efficacy evaluation of washed microbiota transplantation treatment for metabolic related fatty liver disease and its impact on tongue coating microorganisms

**DOI:** 10.3389/fendo.2025.1684173

**Published:** 2025-10-29

**Authors:** Lingui Huang, Siqi Wang, Hao Zhang, Shuo Feng, Haojie Zhong, Junyi Chen, Wenrui Xie, Lei Wu, Tiantian Zhang, Xingxiang He, Juan Yang

**Affiliations:** ^1^ Department of Gastroenterology, The First Affiliated Hospital of Guangdong Pharmaceutical University, Guangzhou, Guangdong,, China; ^2^ Sichuan Integrative Medicine Hospital, Chengdu, Sichuan, China; ^3^ Guangdong Provincial Key Laboratory of Microbial Safety and Health, State Key Laboratory of Applied Microbiology Southern China, Institute of Microbiology, Guangdong Academy of Sciences, Guangzhou, China

**Keywords:** washed microbiota transplantation, metabolic associated fatty liver disease, tongue coating microorganisms, clinical efficacy, biological correlations

## Abstract

**Objective:**

The present study aims to explore the impact of washed microbiota transplantation (WMT) on the tongue microbiota composition of individuals with metabolic-associated fatty liver disease (MAFLD) and elucidate its biological correlations.

**Methods:**

We conducted a comprehensive analysis of hepatic fat deposition and characterized the tongue coating microbiota using 16S rRNA gene sequencing in MAFLD patients before and after undergoing WMT treatment. Furthermore, a MAFLD mouse model was established for additional validation.

**Results:**

At the genus level, significant differences in tongue coating microbiota structure were observed between MAFLD patients and HC. Specifically, *Neisseria* positively correlated with the BARD score, *Porphyromonas* and *Rhodococcus* positively correlated with fat decay, and *Petostreptococcus*, a conditionally pathogenic bacterium, exhibited a significantly higher relative abundance in MAFLD patients compared to HC. Conversely, *Actinomyces* positively correlated with the FIB-4 score, *Megasphaera* negatively correlated with the APRI score, and *Subdoligulum* negatively correlated with low-density lipoprotein levels. Notably, following effective WMT treatment, patients exhibited improved symptoms, with a significant reduction in the relative abundance of *Petostreptococcus* and an increase in potential probiotics such as *Lachnospiraceae* and *Bifidobacterium* in their tongue coating microbiota. Additionally, structural differences in the tongue coating microbiota were identified at the genus level between MAFLD model mice and HC mice. After WMT treatment, the relative abundance of conditionally pathogenic bacteria like *Enterococcus* was significantly decreased in MAFLD model mice.

**Conclusions:**

WMT not only significantly ameliorates liver fat deposition in MAFLD patients but also alters the tongue coating microbial structure associated with disease severity, thereby potentially mitigating adverse patient outcomes.

## Background

Metabolic associated fatty liver disease (MAFLD), a chronic progressive disease characterized by excessive fat accumulation in the liver, has become the most prevalent chronic liver disease globally, with a recent meta-analysis estimating a global prevalence of 38.77% (1). Studies have demonstrated a strong association between MAFLD and the development of liver cirrhosis, liver failure, and even hepatocellular carcinoma. This disease not only poses a significant threat to human health but also places a substantial burden on healthcare systems and the global economy (2).

Lipid metabolism disorder is a primary factor contributing to the development of MAFLD. However, due to the incomplete understanding of its pathogenesis and the absence of clinically approved specific therapeutic drugs, current treatment strategies for MAFLD focus primarily on lifestyle modifications. In addition, pharmacological interventions are often required to address the patient’s underlying conditions. Despite this, most available therapeutic drugs have failed to deliver the expected clinical outcomes (3, 4). Therefore, there remains an urgent need to identify safe and effective treatments for MAFLD, making this a key area of research globally. In recent years, the introduction of the “liver-gut axis” concept has highlighted the role of gut microbiota in the onset and progression of MAFLD. Clinical studies have revealed significant differences in gut microbiota composition between healthy individuals and MAFLD patients, characterized by reduced ecological diversity and an increase in pathogenic bacteria abundance (5). These findings indicate that MAFLD patients experience significant microbial dysbiosis.

The oral microbiota, one of the largest microbial communities in the human body after the gut (6), plays a significant role in systemic inflammation, bacterial infections, and disease progression through its composition and function (7). Studies have shown that patients with liver diseases often exhibit severe oral microbiota imbalances (8). In a clinical study involving 102 patients with liver cirrhosis, severe dysbiosis of the oral microbiota was observed compared to healthy controls, mirroring the dysbiosis seen in the intestinal microbiota (9). Research further indicates that oral microbiota can translocate from the oral cavity to the intestine (10) and may contribute to the development of liver cirrhosis (11). Among the components of the oral microbiota, tongue coating microorganisms have been found to have close associations with various clinical diseases (9). The proportion, density, and diversity of bacterial communities present in tongue coatings are strongly linked to disease formation.

Fecal microbiota transplantation (FMT) involves isolating microbiota from the feces of healthy donors and transplanting the functional bacteria into a patient’s intestine through specialized techniques, with the aim of reshaping the patient’s intestinal microbiota and treating disease (11, 12). FMT has emerged as a breakthrough medical intervention in recent years. An improved version, water-washed microbiota transplantation (WMT), involves using an automated purification system to extract and repeatedly wash the gut microbiota, thereby reducing the risk of FMT-related adverse effects. WMT has already been investigated in various metabolic diseases, including dyslipidemia (13, 14), obesity (15), diabetes (16), and hypertension (17).

We hypothesize that WMT may offer clinical benefits for patients with MAFLD and that a correlation exists between the microbiota present on the tongue coating of MAFLD patients and the efficacy of WMT treatment. To explore this hypothesis, we employ a combination of animal and human studies to evaluate the potential therapeutic effects of WMT on MAFLD.

## Methods

### Inclusion criteria for MAFLD

This study included MAFLD inpatients aged ≥18 years who were admitted to the First Affiliated Hospital of Guangdong Pharmaceutical University between January 2017 and December 2022. MAFLD was diagnosed based on international consensus criteria (18). The exclusion criteria for patients were as follows: (1) Use of antibiotics within the past month; (2) Presence of dental caries or periodontal disease; (3) Severe heart, lung, or kidney diseases; (4) Coexisting liver diseases; (5) Serious lack of medical records. This study was approved by the Ethics Committee of the First Affiliated Hospital of Guangdong Pharmaceutical University (#2021-13), and written informed consent was obtained from all participants.

### Sample collection

#### Person: serum, tongue coating

Medical record data for the WMT group were collected at three time points: before treatment (baseline), before each WMT procedure, and after the final WMT treatment. Data included laboratory parameters such as BMI, age, gender, liver enzymes, fasting insulin, blood lipids, fasting blood glucose, and liver imaging (CT, ultrasound, and elastography). Sample collection from patients followed a standardized protocol. Patients were instructed to avoid eating and brushing their teeth for at least 1 hour prior to sampling. After rinsing their mouths with physiological saline, a sterile cotton swab was used to collect samples from the middle of the tongue. The swab was placed into an RNase-free Eppendorf tube, 1 mL of phosphate buffer solution (PBS) was added, and the mixture was stirred. The sample was centrifuged at 4000 rpm for 20 minutes, after which the supernatant and precipitate were separately stored in sterile tubes. All samples were kept at -80 °C until further analysis.

#### Mice: tongue coating and liver samples

Mouse tongue coating collection: (1) Anesthesia: Administer 0.05 mL of Shu Tai via intramuscular injection per mouse. (2) Sample Collection: Using tweezers, gently pull the mouse’s tongue out of its mouth, quickly sever the tongue at the base, and place it in a sterile tube. All samples were stored at -80 °C until analysis. The entire procedure was completed within 1 minute after the mouse’s death. The mouse liver collection procedure follows these steps: (1) Anesthesia: Administer 0.05 mL of Shu Tai via intramuscular injection per mouse. (2) Dissection: Incise the abdominal skin to fully expose the thoracic and abdominal cavities. (3) Cardiac Perfusion: Locate the abdominal aorta and perform an incision. Using a syringe, inject 30 mL of physiological saline into the heart from the apex until the effluent becomes lighter in color. (4) Liver Tissue Extraction: Identify the liver, gently detach it with tweezers, and carefully remove the intact organ. (5) Tissue Processing: Remove any surrounding ligaments and the gallbladder. Divide the liver into three sections, preserving one relatively intact portion for pathological analysis, which should be stored in an Eppendorf tube containing 4% PFA fixative. (6) Pathological Staining: Conduct staining procedures for pathological examination on the preserved liver tissue.

### WMT

Questionnaire surveys, physical examinations, blood and stool tests, and other laboratory screenings were conducted for all healthy fecal donors aged 18 to 25 years. This study employed the Nanjing Consensus washing bacterial transplantation method (19). In a biosafety level 2 laboratory, bacterial suspensions were prepared with the assistance of trained professionals using disposable sterile materials. To prepare the washed microbial community, a homogeneous fecal suspension was created by mixing 100 g of feces with 500 mL of 0.9% saline solution in a 1:5 ratio. This mixture was then subjected to microfiltration using an automatic purification device (Gen^FM^Ter, FMT Medical). The microbial precipitate was washed three times, after which 100 mL of saline solution was added to create the bacterial suspension. This suspension was maintained in a water bath at a constant temperature of 37 °C and injected within 1 hour to prevent contamination or compositional changes.

Based on the physical condition and willingness of each MAFLD patient, the washed bacterial suspension was administered into the patient’s intestine via either the upper or lower digestive tract (120 mL per day for 3 consecutive days). This study followed the “Three Three Principles” therapy, where one treatment course consisted of continuous injection of the washing bacterial suspension for 3 days, followed by three consecutive months of treatment (i.e., three courses), and another course after a 3-month interval to stabilize bacterial colonization. The total treatment duration was 6 months. All patients underwent at least one WMT procedure and completed follow-up assessments by October 31, 2022.

### Mouse experiment

#### Establishment of MAFLD mouse model

This study utilized a methionine and choline-deficient (MCD) diet to freely feed C57BL/6 mice (Guangdong Medical Animal Experimental Center) for 4 weeks, thereby establishing a MAFLD mouse model (17). The animal experiments received approval from the Animal Ethics Committee of the First Affiliated Hospital of Guangdong Pharmaceutical University (#2022-14) and were conducted in accordance with the guidelines for reporting *in vivo* experiments involving animals.

#### WMT experiment in mice

Preparation of bacterial solution follows these steps: (1) Sterile Procedure: All operations should be conducted in a sterile room to maintain a contamination-free environment. (2) Sample Collection: Collect fecal samples separately from MAFLD mice and healthy mice, gathering 2 or more fresh fecal samples from each mouse, and label them appropriately. (3) Fecal Suspension: Soak the collected feces in a 0.1 g/mL physiological saline solution for at least 10 minutes, then homogenize the mixture. (4) Centrifugation: Centrifuge the homogenized suspension at 1000 rpm for 3 minutes and collect the supernatant. Gavage Procedure: MAFLD mice were divided into two groups: one group received the bacterial solution from mice with more severe disease, while the other group received the bacterial solution from healthy mice. Gavage was administered at a dose of 0.2 mL per animal, given once every other day for four consecutive weeks.

### DNA extraction and 16S rRNA

Total DNA from the tongue coating of MAFLD patients and the tongues of MAFLD mice was extracted according to the E.Z.N.a^®^ protocol using the Soil DNA Kit (Omega BioTek, Norcross, GA, USA). The quality and concentration of all DNA samples were evaluated using a NanoDrop 2000 spectrophotometer (Thermo Fisher Scientific, Wilmington, DE, USA). The V3-V4 fragment of the bacterial 16S rRNA gene was amplified by PCR using primers 806R and 338F. The sequences of the primers were as follows: 5’-GACTACHVGGGTWTCTAAT-3’ (338F) and 5’-ACTCCTACGGGAGGCAGCAG-3’ (806R). The PCR conditions included an initial denaturation at 95 °C for 30 seconds, followed by 27 cycles of 30 seconds at 55 °C, 45 seconds at 72 °C, and a final extension at 72 °C for 5 minutes. The PCR reaction mixture contained: 4 μL of 5 × TransStart FastPfu buffer, 2 μL of 2.5 mM deoxyribonucleoside triphosphates (dNTPs), 0.8 μL of 5 μM primers, 0.4 μL of TransStart FastPfu DNA polymerase, and 10 ng of extracted DNA, with the final volume adjusted to 20 μL with ddH2O. Agarose gel electrophoresis was performed to verify the size of the PCR amplicon. Sequencing was conducted using the Illumina MiSeq PE300 platform (Shanghai MajorbioBio, China).

### H.E. staining

HE staining: Prepared slices were placed in a fully automated staining machine for HE staining. After the staining process was complete, the slices were sealed for further analysis.

### Data statistical analysis

Statistical analyses were performed using GraphPad Prism 9.0.0 and SPSS 25.0 software. Changes in clinical indicators of MAFLD patients before and after WMT were presented as differences using a self-matching design. Continuous variables were first assessed for normality. mean ± standard deviation (M ± SD) was used to describe continuous variables with a normal distribution, analyzed using a one-sample t-test. For non-normally distributed continuous variables, the median (interquartile range) was reported and analyzed using the one-sample rank sum test. Categorical variables were represented by frequency and percentage. A bilateral p-value of < 0.05 was considered statistically significant, with *p <* 0.05 denoted by *, *p <* 0.01 denoted by **, and *p <* 0.001 denoted by ***.

## Results

### Evaluation of clinical efficacy of WMT in the treatment of MAFLD

#### Comparative analysis of baseline level information for the samples included in this study

A total of 175 subjects participated in this study, consisting of 76 MAFLD patients, 66 NMD patients, and 33 healthy control (HC) volunteers. The baseline physiological characteristics of all participants are summarized in **Table 1**. Significant differences (*p <* 0.05) were observed in multiple clinical indicators between the MAFLD group and the HC group. These indicators included body mass index (BMI), white blood cell count (WBC), fat decay (FD), liver hardness (LH), total cholesterol (TC), triglycerides (TG), high-density lipoprotein (HDL), fasting blood glucose (FBG), homeostasis model assessment-insulin resistance (HOMA-IR), alanine aminotransferase (ALT), aspartate aminotransferase (AST), albumin (ALB), gamma-glutamyl transferase (GGT), MAFLD fibrosis score (MFS), aspartate aminotransferase-to-platelet ratio index (APRI), and fibrosis 4 Score, (FIB-4). In contrast, no significant differences were noted in other aspects. These clinical data indicate that the MAFLD patients in this study require further intervention to address the ongoing progression of the disease. Furthermore, the non-MAFLD other diseases (NMD) patient sample served as a reference group for subsequent analyses.

**Table 1:** Baseline physiological characteristics of all participant samples.

Project		Group		
		MAFLD	NMD	HC
Basic information	Age (Years)	53.04 ± 15.38	43.24 ± 14.73	44.92 ± 14.35
	M/F (n/n)	35/41	32/34	14/19
	BMI (kg/m^2^)	26.42 ± 4.19 ***	21.06 ± 3.17	19.75 ± 1.65
	WBC (10^9^/L)	6.96 ± 2.10 ***	6.29 ± 1.97 **	5.24 ± 1.19
	RBC (10^9^/L)	4.51 ± 0.69	4.37 ± 0.65	4.42 ± 0.50
Fibro touch	FD (dB/m)	284.54 ± 30.38 ***	215.00 ± 34.53	219.56 ± 15.81
	LH (kPa)	0.96 ± 0.67 ***	5.73 ± 1.49	6.22 ± 1.11
Glycolipid index	TG (mmol/L)	2.04 ± 1.55 **	0.92 ± 0.37	1.03 ± 0.32
	TC (mmol/L)	5.35 ± 1.20 *	4.46 ± 1.05	4.98 ± 0.88
	HDL (mmol/L)	1.13 ± 0.27 ***	1.32 ± 0.33	1.38 ± 0.28
	LDL (mmol/L)	3.29 ± 1.06 *	2.73 ± 0.86 **	3.16 ± 0.75
	FBG (mmol/L)	5.45 ± 1.71 *	4.37 ± 0.45	4.41 ± 0.61
	HOMA-IR (20)	3.06 ± 2.55 ***	1.27 ± 0.88	0.73 ± 0.34
Liver function related indexes and scores	ALT (U/L)	42.83 ± 3.52 **	21.36 ± 30.09	14.62 ± 5.46
	AST (U/L)	29.46 ± 22.94 **	23.97 ± 18.31	19.43 ± 4.98
	ALB (g/L)	42.83 ± 3.52	41.47 ± 4.05	46.32 ± 3.53
	GGT (U/L)	59.19 ± 66.82 *	22.82 ± 16.94	15.10 ± 5.92
	DB	4.62 ± 1.47	9.54 ± 41.80	4.82 ± 1.83
	IB	8.16 ± 3.32	10.72 ± 14.86	8.36 ± 2.34
Disease severity related score	MFS (21)	2.70 ± 0.68 ***	1.91 ± 0.66	1.73 ± 0.46
	APRI (21)	0.33 ± 0.28 *	0.41 ± 0.83	0.24 ± 0.07
	FIB-4 (22)	0.31 ± 0.30 *	0.62 ± 2.26	0.28 ± 0.14

MAFLD, metabolic associated fatty liver disease; NMD, Non-MAFLD other diseases; HC, health control; M/F, mela/female; BMI, basic measuring instrument; WBC, white blood cell; RBC, red blood cell; FBG, fasting blood glucose; FD, Fat decay; LH, liver hardness; TC, total cholesterol; TG, triglyceride; HDL, high-density lipoprotein; LDL, low-density lipoprotein; HOMA-IR, homeostasis model assessment-insulin resistance; ALT, alanine aminotransferase; AST, aspartate aminotransferase; ALB, albumin; GGT, gamma-glutamyl transferase; DB, direct bilirubin; IB, iIndirect bilirubin; MFS, MAFLD fibrosis score; APRI, aspartate aminotransferase-to-platelet ratio Index; FIB-4, Fibrosis 4 Score; *, *p <* 0.05 vs. HC; **, *p <* 0.01 vs. HC; ***, *p <* 0.001 vs. HC.

### Clinical efficacy evaluation of WMT treatment for MAFLD

**Figure 1:**
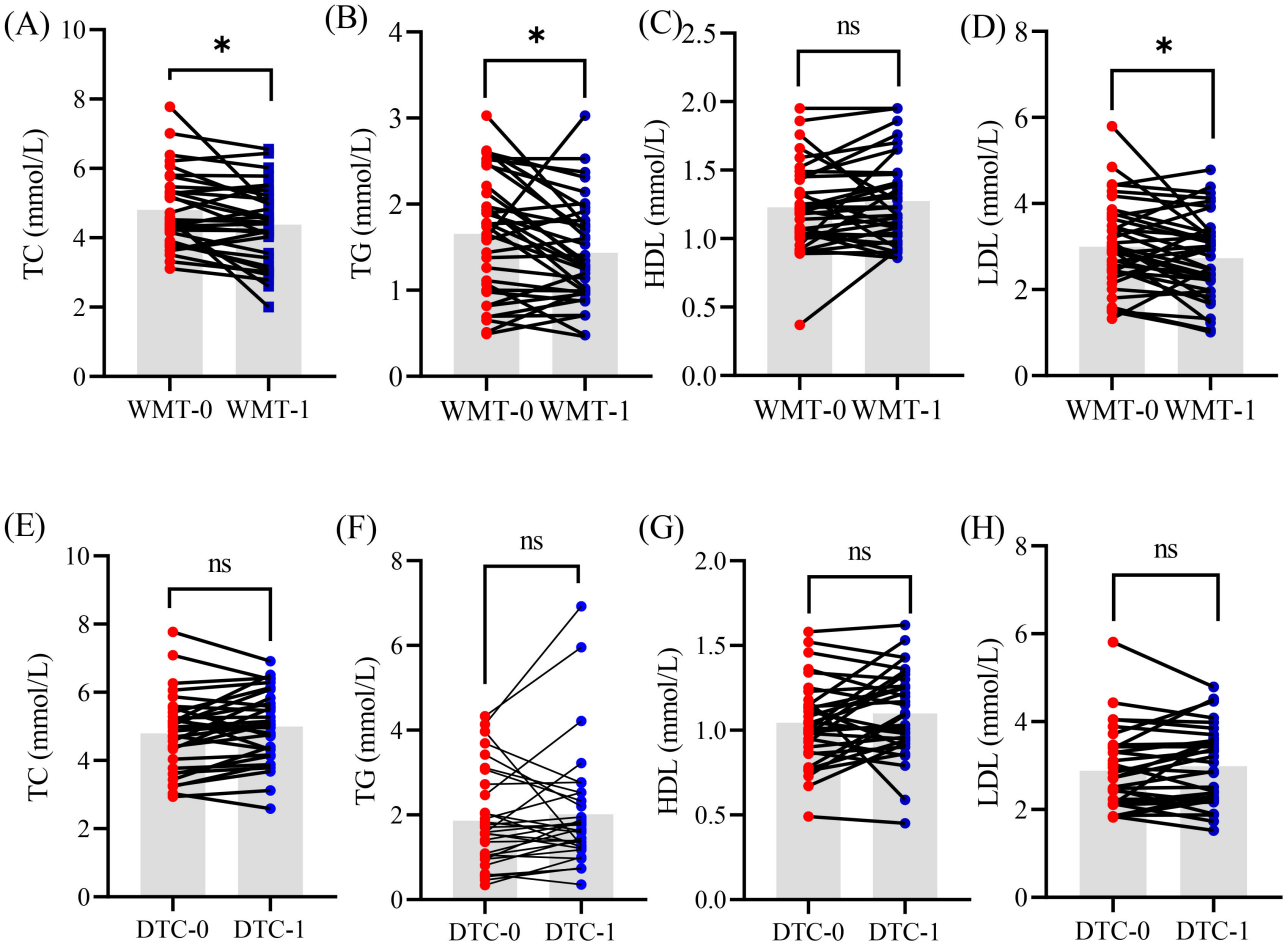
The glucose and lipid indicators of patient MAFLD undergoing clinical treatment with WMT **(A–D)** and DTC **(E–H)** methods. WMT, washed microbiota transplantation; TC, total cholesterol; TG, triglyceride; HDL, high-density lipoprotein; LDL, low-density lipoprotein; DTC, drug therapy control; 0, baseline level before treatment; 1, 1 treatment cycle; *, *p <* 0.05; ns, no significant.

To assess the clinical efficacy of WMT for treating MAFLD, the patients in this study were randomly assigned to two groups: the WMT group (n = 43) and the drug therapy control (DTC) group (n = 33), in which the medication of group DTC was completely in accordance with the “guidelines for the prevention and treatment of nonalcoholic fatty liver disease” (23). Concurrently, various clinical indicators were collected from both groups prior to treatment. Based on the statistical analysis, glucose and lipid indicators closely associated with MAFLD were compiled and visualized (**Figure 1**).

After one course of WMT treatment, MAFLD patients demonstrated significant reductions in TC, TG, and LDL (*p <* 0.05), while HDL levels remained unchanged. These findings suggest that WMT can effectively improve glucose and lipid metabolism in patients with MAFLD to some extent. In contrast, no significant changes were observed in these indicators among patients in the DTC group during the same treatment period. This evidence indicates that WMT treatment may have a beneficial impact on MAFLD in clinical practice.

### Long-term efficacy evaluation of WMT treatment for MAFLD

**Figure 2:**
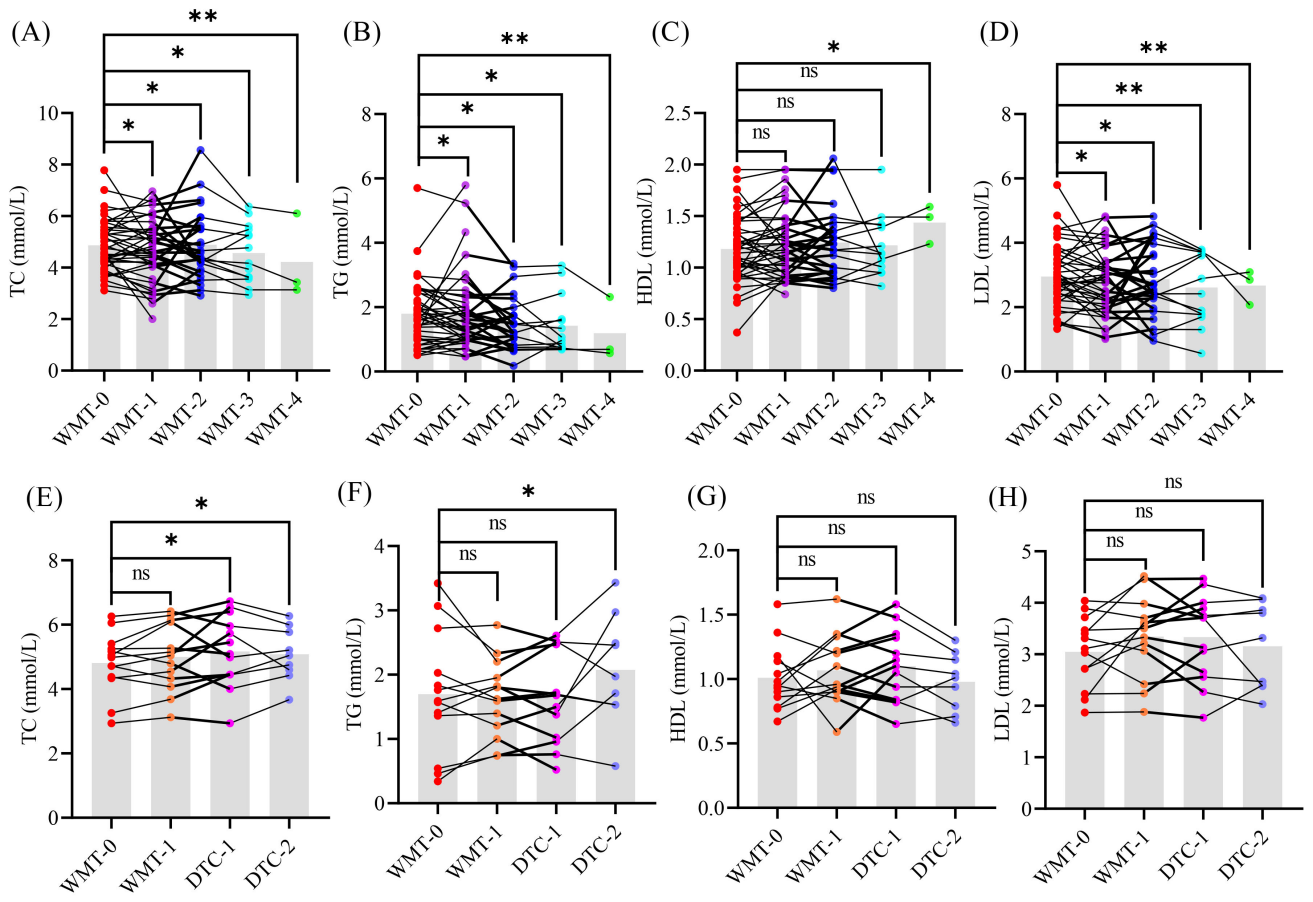
The glucose and lipid indicators of patients MAFLD **(A–D)** and NMD **(E–H)**. WMT, washed microbiota transplantation; TC, total cholesterol; TG, triglyceride; HDL, high-density lipoprotein; LDL, low-density lipoprotein; DTC, drug therapy control; 0, baseline level before treatment; 1 - 4, different treatment cycle; *, *p <* 0.05; **, *p <* 0.01; ns, no significant.

To further evaluate the clinical efficacy of long-term WMT treatment for MAFLD, we conducted a statistical analysis of the glucose and lipid indicators in 43 MAFLD patients who participated in WMT (**Figure 2**). Among these patients, 27 underwent 2 courses of WMT, 14 completed 3 courses, and 3 received 4 courses of WMT. Additionally, we included clinical data from NMD patients for comparative analysis.

As illustrated in the results graph, an increasing number of treatment cycles corresponded with a significant downward trend in the levels of TC, TG, and LDL compared to the baseline levels (WMT-0) in the MAFLD group. Conversely, HDL levels exhibited a significant upward trend, suggesting that long-term WMT treatment can substantially enhance glucose and lipid metabolism in MAFLD patients.

Interestingly, no significant differences were observed in glucose and lipid indicators between NMD patients and their baseline levels, nor were any changes noted following one cycle of WMT treatment. This indicates that WMT does not cause disturbances in glucose and lipid indicators, further supporting its safety and efficacy in managing MAFLD.

### Relationship between tongue coating microorganisms and MAFLD

#### Differences in tongue coating microbiota between MAFLD patients and HC

**Figure 3:**
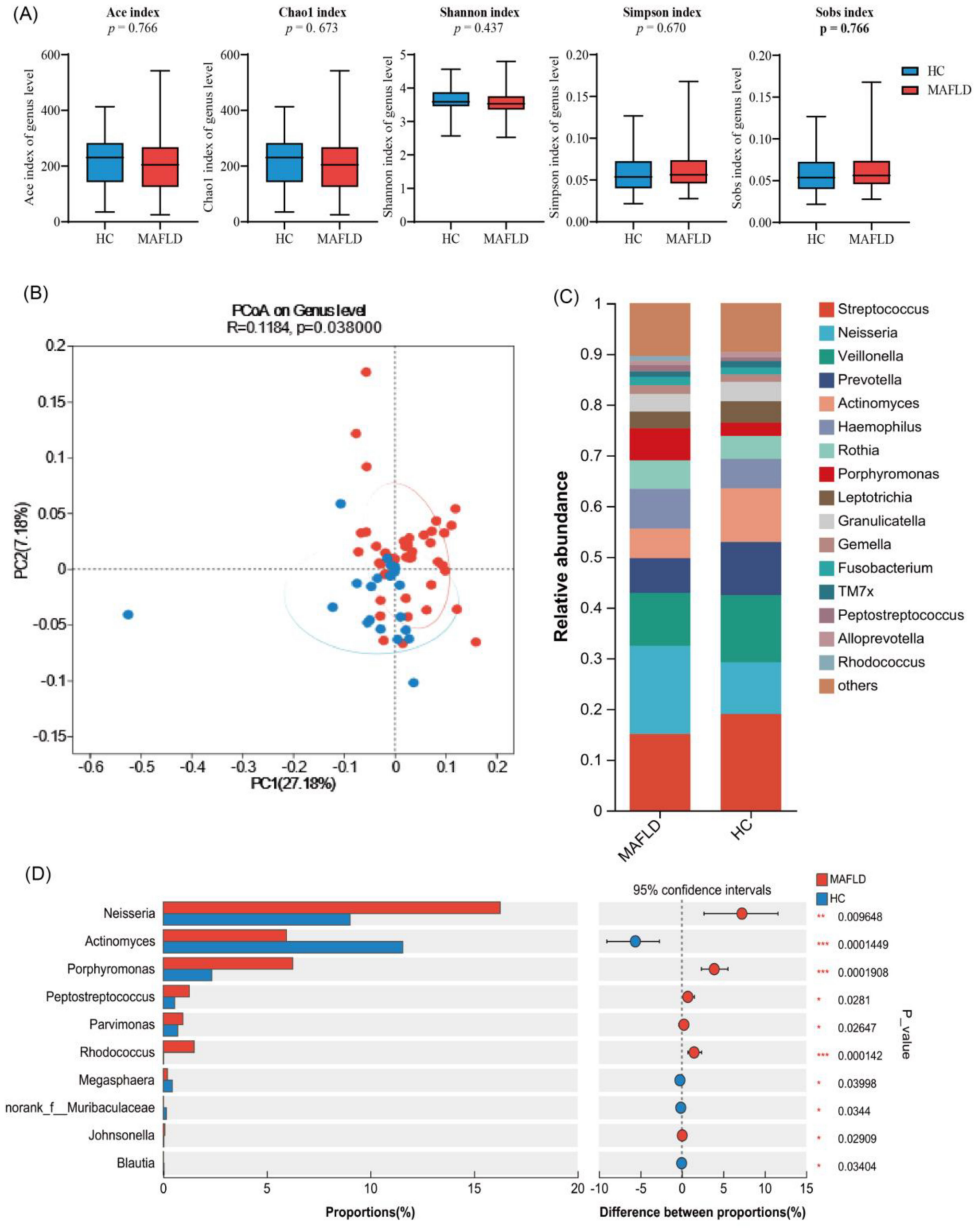
Differential analysis of tongue microbiota between MAFLD patients and healthy individuals based on 1 6S rRNA sequencing. **(A)** Ace, Chao1, Sobs, Simpson, and Shannon indices explain the alpha diversity of tongue coating microbiota; **(B)** Display the β diversity of tongue coating microbiota in MAFLD patients (n = 56) and HC group (n = 26) through PCoA plot; **(C)** The bar chart displays the differences in genus levels between two groups of tongue coating bacteria; **(D)** Wilcoxon rank sum test bar chart at the genus level.

To investigate the tongue microbiota of MAFLD patients, we analyzed samples from 56 MAFLD patients and 26 healthy individuals. The species richness and diversity of the two microbial communities were further assessed (**Figure 3**). Our results indicated no statistically significant differences in the ACE, Chao1, Shannon, and Simpson indices between the two groups (**Figure 3A**), suggesting that microbial community richness and diversity in the tongue coatings were comparable between the two populations.

Additionally, we performed genus-level clustering analysis using Principal Coordinates Analysis (PCoA) on the two sample groups (**Figure 3B**), which demonstrated no significant differences in microbial community structure between them. Notably, further analysis revealed that the Wilcoxon rank sum test indicated significantly higher relative abundances of *Neisseria*, *Porphyromonas*, *Rhodococcus*, and *Peptoniphilus* in the MAFLD group compared to the healthy controls. Conversely, *Actinobacteria*, *Megasphaera*, *Blautia*, and *Subdoligranulum* exhibited significantly higher abundances in the healthy population (**Figures 3C, D**). These findings indicate distinct differences in the tongue coating microbiota between MAFLD patients and HC group.

#### Correlation analysis between tongue coating microorganisms and clinical metabolism in MAFLD patients

**Figure 4:**
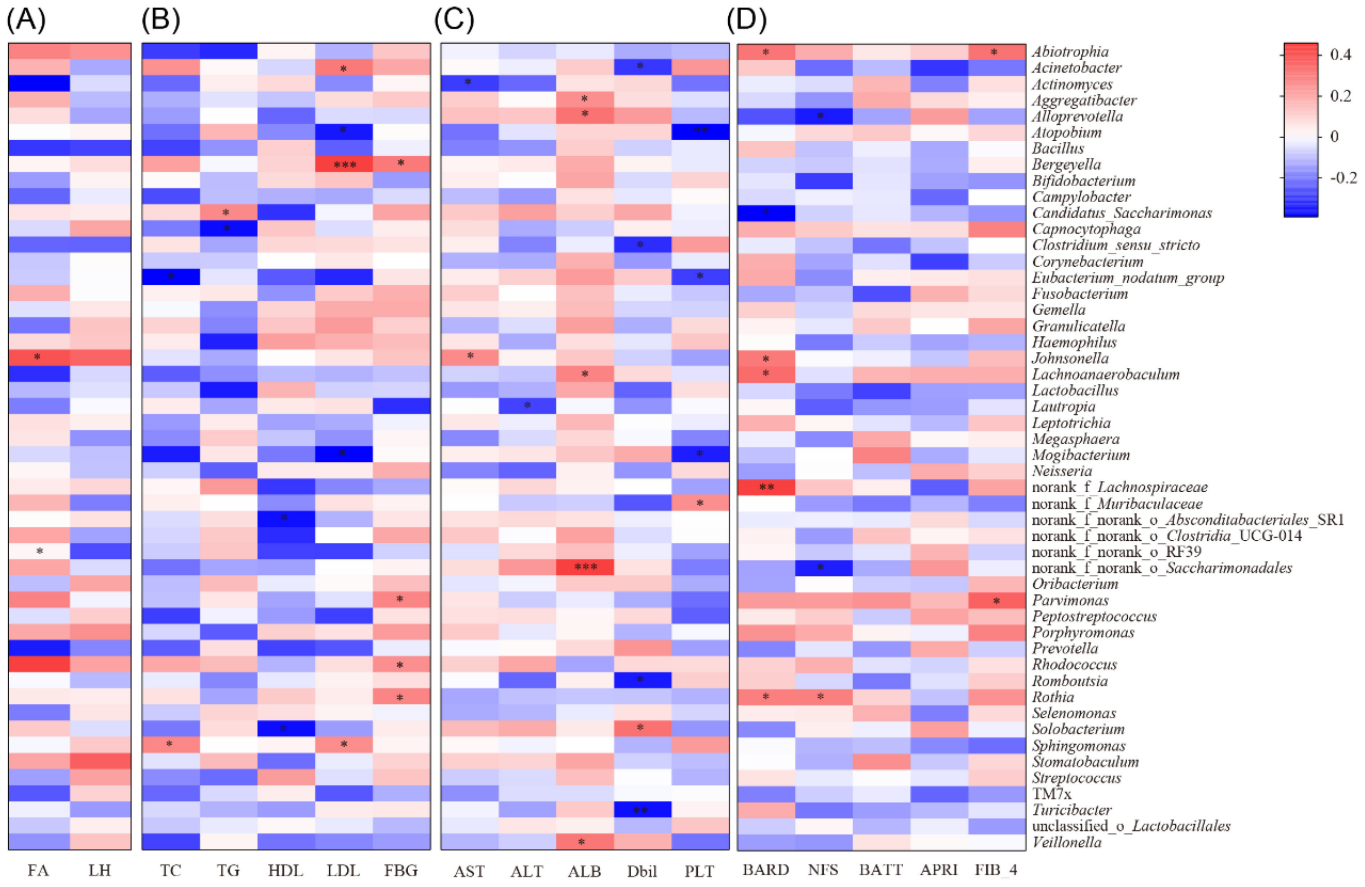
Correlation analysis between different clinical indicators and tongue coating microbiota in MAFLD patients. **(A)** Correlation heatmap of FA and LH relative to the abundance and composition of tongue coating microbiota in MAFLD patients; **(B)** Correlation heatmap between glucose and lipid indicators (TC, TG, HDL, LDL and FBG) and the abundance and proportion of tongue microbiota in MAFLD patients; **(C)** Correlation heatmap of liver function indicators (AST, ALT, ALB, Dbil, PLT) between the abundance and proportion of tongue microbiota genera in MAFLD patients; **(D)** Correlation heatmap of disease severity related scores (BARD, NFS, BATT, APRI, FIB-4) between the abundance and proportion of tongue microbiota genera in MAFLD patients; *, *p <* 0.05;**, *p <* 0.01;***, *p <* 0.001.

Further analysis revealed that *Rhodococcus* and *Johnsonella* in the tongue microbiota of MAFLD patients were positively correlated with the liver fat attenuation index (*p <* 0.05, as shown in **Figure 4A**). Additionally, TC and LDL levels in MAFLD patients were positively correlated with *Sphingomonas*, while *Bergeyella* exhibited a positive correlation with LDL. Conversely, *Capnocytophaga* demonstrated a negative correlation with TG, and *Atopobium* showed a negative correlation with LDL. Moreover, *Rothia* and *Rhodococcus* were positively correlated with fasting blood glucose (FBG) (*p <* 0.05, **Figure 4B**).

#### Correlation analysis between tongue coating microorganisms and liver function in MAFLD patients

In addition, we observed a positive correlation between *Johnsonella* and AST, as well as a positive correlation between *Saccharimondales* and *Veillonella* with ALB. Conversely, *Atopobium* was negatively correlated with PLT (*p <* 0.05, **Figure 4C**). Furthermore, we found that the genus *Rothia* was positively correlated with the AST to APRI scores, while *Neisseria* exhibited a positive correlation with the BARD score. Additionally, there was a positive correlation (*p <* 0.05, **Figure 4D**) between *Parvimonas* and *Abiotrophia* with the FIB-4 score.

### The effect of WMT on tongue microbiota in MAFLD patients

#### Impact of WMT on tongue coating microecology in MAFLD patients

**Figure 5:**
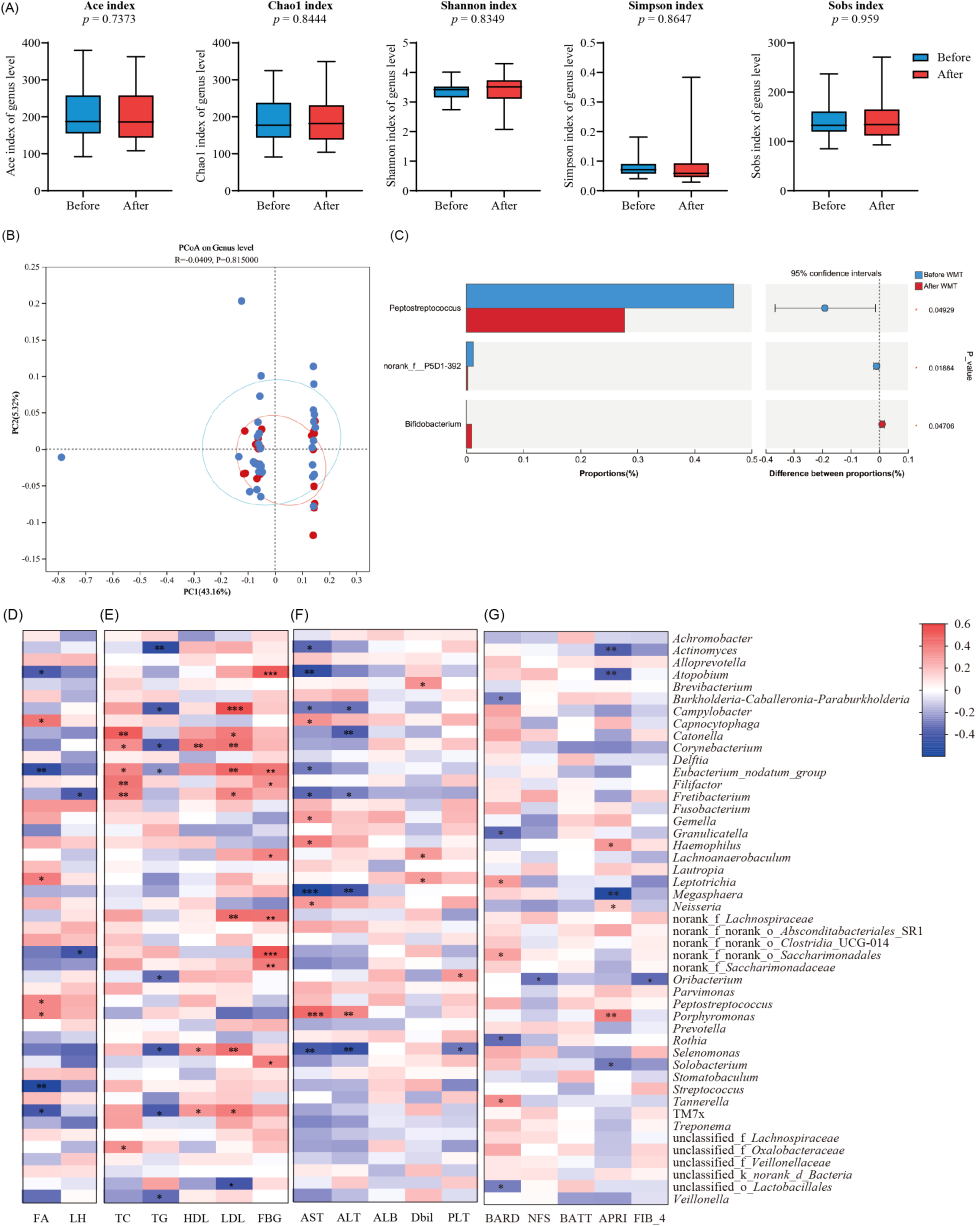
The effect of WMT on tongue microbiota in MAFLD patients. **(A)** Box plots comparing the alpha diversity indices (Ace, Chao1, Sobs, Simpson, and Shannon) of tongue coating microbiota in MAFLD patients before and after WMT; **(B)** Differentiate the β diversity of two groups of microorganisms through POCA plot **(C)** and plot the horizontal differences in the genera of two groups of tongue coating microorganisms; **(D)** Correlation heatmap between the abundance and proportion of tongue microbiota genera and fat attenuation and liver hardness in MAFLD patients after WMT; **(E)** Correlation heatmap of glucose and lipid indicators (TC, TG, HDL, LDL, FBG) at the level of abundance and proportion of tongue microbiota in MAFLD patients after WMT; **(F)** Correlation heatmap of liver function indicators (AST, ALT, ALB, Dbil, PLT) between the abundance and proportion of tongue microbiota genera in MAFLD patients after WMT; **(G)** Correlation heatmap of disease severity related scores (BARD, NFS, BATT, APRI, FIB-4) between the abundance and proportion of tongue microbiota genera in MAFLD patients after WMT; *, *p <* 0.05;**, *p <* 0.01;***, *p <* 0.001.

This study found that the alpha diversity of the tongue microbiota in MAFLD patients decreased after WMT treatment, with the exception of the Shannon index, and there were no significant changes in the ACE, Sobs, or Simpson indices (**Figure 5A**). PCoA analysis also revealed no significant alterations (**Figure 5B**), indicating that the microbial community structure remained relatively stable. Furthermore, the Wilcoxon rank sum test indicated a significant decrease in the relative abundance of opportunistic pathogens, such as *Peptostreptococcus*, in MAFLD patients following WMT. Conversely, the abundance of potential probiotics, including *Lachnospiraceae* and *Bifidobacterium*, increased significantly (*p <* 0.05, **Figure 5C**).

Further analysis revealed that *Porphyromonas*, *Leptotrichia*, and CO2-oxidizing bacteria in the tongue microbiota of MAFLD patients post-WMT were positively correlated with fat attenuation. In contrast, *Streptococcus* and other bacteria were positively associated with the fat attenuation burden in these patients (*p <* 0.05, **Figure 5D**). Additionally, the genera *Catonella*, *Fretibacterium*, and *Filifactor* showed positive correlations with TC indicators in patients, while *Campylobacter*, *Corynebacterium*, and *Eubacterium* were positively correlated with both HDL and LDL. Moreover, *Atonobium*, *Saccharimonadales*, and *Lachnoanaerobaculum* were positively associated with FBG levels (*p <* 0.05, **Figure 5E**).

#### Correlation analysis between tongue coating microorganisms and liver function in MAFLD patients treated with WMT

Through the analysis of liver function indicators, we found that the genera *Haemophilus* and *Porphyromonas* were positively correlated with liver enzyme levels (ALT, AST), while *Oribaterium* showed a positive correlation with ALB (*p <* 0.05, **Figure 5F**). Additionally, *Porphyromonas* and *Haemophilus* were positively correlated with disease severity scores, whereas *Megasphaera* exhibited a negative correlation with APRI scores (*p <* 0.05, **Figure 5G**).

### The effect of WMT on MAFLD mice

#### Effects of WMT on the microecology of tongue coating in MAFLD mice

**Figure 6:**
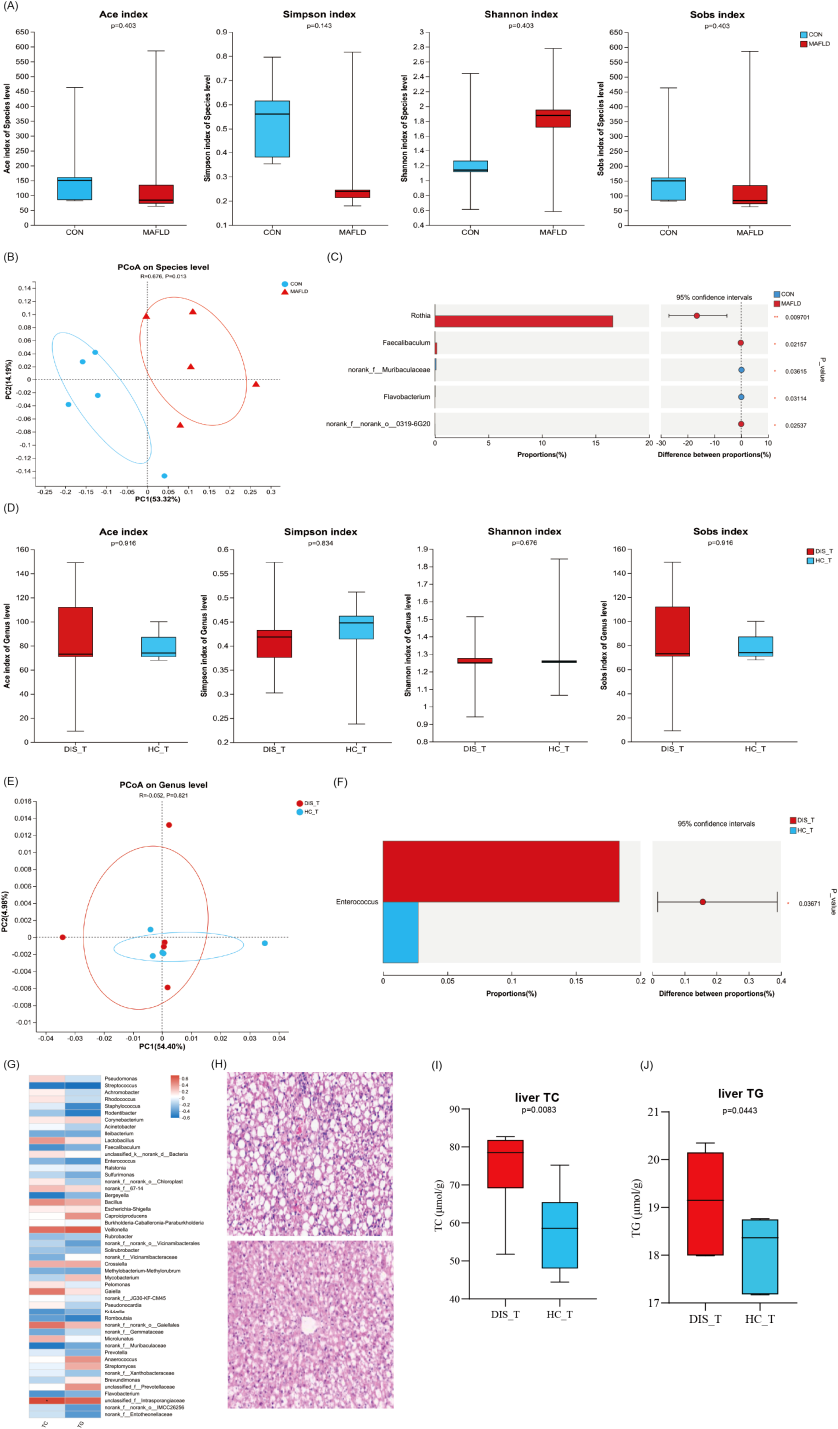
The effect of WMT on MAFLD mice. Differential analysis of tongue coating microbiota between healthy mice vs. MAFLD mice **(A–C)** and MAFLD mouse disease group vs. healthy group after WMT **(D–F)**; **(G)** Heatmap of TC, TG and tongue coating microbiota in MAFLD mice after WMT; **(H)** H.E. staining of liver tissue; The levels of TC **(I)** and TG **(J)** in the liver of MAFLD mice.

Animal experiments revealed significant differences in the tongue coating microbiota between MAFLD mice and healthy control (CON) mice (**Figures 6A–C**). The results of the PCoA for beta diversity indicated no overlap in the microbial communities of the tongue coating at the species level between the MAFLD and CON groups (**Figure 6B**), highlighting alterations in the composition and structure of the tongue coating microbiota in MAFLD mice. Further analysis using Wilcoxon rank sum tests at the genus level demonstrated that the abundances of the genera *Rothia* and *Faecalibaculum* were significantly higher in the MAFLD group, while the abundances of *Muribaculaceae* and *Flavobacterium* were significantly elevated in the CON group (*p <* 0.05, **Figure 6C**). These findings indicate a substantial difference in the tongue coating microbiota between the two groups.

Following FMT treatment, no significant statistical differences were observed in the alpha diversity indices (ACE, Chao1, Shannon) of the tongue microbiota between the group receiving severe fatty liver fecal microbiota transplantation (DIS_T) and the group receiving healthy fecal microbiota (HC_T) (**Figure 6D**). This indicates that there were no significant differences in the richness and diversity of the tongue microbiota between the two groups of mice. Furthermore, beta diversity analysis of the two groups of MAFLD mice also revealed no significant differences in the microbial structure of the tongue coating (**Figure 6E**). However, the Wilcoxon rank sum test at the genus level identified a higher abundance of the opportunistic pathogenic bacterium *Enterococcus* in the disease microbiome (*p <* 0.05, **Figure 6F**). Additional analysis showed that the levels of TC and TG in MAFLD mice following FMT were positively correlated with the genera *Veillonella*, *Intrasporangiaceae*, *Gaielle*, and *Bacillus*, while negatively correlated with *Streptococcus*, *Faecalibaculum*, *Bergeyella*, *Kribbella*, and *Romboutsia* (*p <* 0.05, **Figure 6G**).

#### Effects of WMT on the liver of MAFLD mice

Histological analysis of the livers from mice revealed that the liver lobule structure in the DIS_T group exhibited more severe damage, with a higher degree of hepatic cell steatosis compared to the HC_T group. Additionally, focal necrosis was significantly more prevalent in the livers of DIS_T mice than in those of HC_T mice (**Figure 6H**). Furthermore, the levels of TC and TG per gram of liver in DIS_T mice treated with FMT were significantly elevated compared to those in HC_T mice (*p <* 0.05, **Figures 6I, J**).

## Discussion

### Discussion on the clinical treatment effect of WMT on MAFLD

In recent years, numerous studies have demonstrated that regulating gut microbiota through FMT can be an effective treatment for MAFLD. A randomized controlled trial investigating the use of FMT for MAFLD treatment found that FMT can significantly reduce liver fat deposition and alleviate disease symptoms by improving gut microbiota dysbiosis (24). However, there is a relative paucity of studies examining the clinical efficacy of WMT, an advanced form of FMT, specifically in the context of MAFLD. Due to the small sample size in our previous research, we plan to expand our sample size in this study to further evaluate the clinical efficacy of WMT on MAFLD. The findings of this study align with previous research, indicating that WMT can effectively reduce the hepatic steatosis index in MAFLD patients. In a separate clinical randomized controlled study involving 87 obese patients, participants were divided into two groups, with one group receiving FMT and the other a placebo. After the same treatment course, the FMT group showed significant improvements in fat ratio and metabolic disorders (25). Our team has also conducted a randomized controlled clinical trial that demonstrated FMT’s ability to reduce fat accumulation in the liver by reshaping gut microbiota dysbiosis, thus improving therapeutic outcomes for MAFLD patients (25). Notably, its clinical efficacy appears to be higher in lean MAFLD patients compared to their obese counterparts (24). These findings suggest that WMT may represent a promising new approach for the treatment of MAFLD.

However, some studies have reported that while FMT can improve gut microbiota and barrier function in patients with MAFLD, it does not significantly alter liver fat deposition (26). In this study, we observed that the recovery rate of the liver fat attenuation index in MAFLD patients was 21.21% after the first course of treatment, 40.91% after the second course, but only 30% after the third course. This decline may be attributed to the extended interval between the third and fourth WMT treatments, which likely led to a reduction in the diversity and abundance of the gut microbiota, thereby diminishing therapeutic efficacy. Previous studies have suggested that the effectiveness of FMT is closely related to various factors, including the characteristics of the donor’s microbiota, the recipient’s intestinal colonization resistance, and the frequency of FMT treatments (27, 28). Consequently, we hypothesize that short-term, multiple WMT sessions may provide patients with a healthier and higher-quality microbiota characterized by greater richness and diversity. This enhancement could be a critical factor contributing to the significant improvement in fat deposition observed in MAFLD patients. It is also important to note that dysregulation of glucose and lipid metabolism is a key feature in the progression of MAFLD (27). In our study, we found that with increasing treatment cycles, TC, TG, and LDL levels significantly decreased in MAFLD patients, indicating improvements in glucose and lipid metabolism following WMT. Notably, no serious adverse events were reported during the course of this study, consistent with findings from other clinical studies indicating that FMT does not lead to serious adverse outcomes. This suggests that WMT is also a safe intervention.

### Discussion on the correlation between tongue coating microorganisms and MAFLD

As a significant component of the oral microbiota, tongue coating microbiota has become a focal point for researchers in microbiology, both domestically and internationally. The density, diversity, and changes in the tongue coating microbiota can reflect the physiological status of the human body and the alterations in its microbiota composition (27, 28).In this study, we found that the abundance of *Porphyromonas* in the tongue microbiota of MAFLD patients was significantly higher than that in healthy individuals. Similar findings have been reported in a study, indicating a significant association between *Porphyromonas* and the occurrence and progression of MAFLD (29). Additionally, the study by Masato (30) identified a higher proportion of *Porphyromonas* in MAFLD patients, positively correlating with disease severity. This suggests that *Porphyromonas* could serve as a potential biomarker for MAFLD.

The composition of microorganisms in the tongue coating of healthy individuals remains relatively stable. Previous scholars have reported on the specific proportions of various bacteria, including *Streptococcus salivarius* (20%), *Streptococcus pyogenes* (4%), *Streptococcus thermophilus* (8%), and *Neisseria* (0.05%) (31, 32). In contrast, our study observed that the abundance of the *Neisseria* genus in MAFLD patients significantly exceeded this baseline, indirectly confirming the existence of tongue coating microbiota dysbiosis in this population.

Furthermore, we identified significantly lower abundances of *Pseudomonas aeruginosa* and *Subdoligulum* in MAFLD patients, aligning with findings reported by Diaz (33), which noted that these genera were less abundant in the gut microbiota of MAFLD patients compared to healthy controls. Collectively, these results suggest that *Porphyromonas*, *Pseudomonas aeruginosa*, and *Subdoligulum* may serve as potential biomarkers for MAFLD. Additionally, our animal studies confirmed significant differences in tongue coating microbiota between MAFLD and healthy controls, indicating that MAFLD may have identifiable biomarkers within the tongue coating microbiota.

In addition, multiple microbial communities in the tongue coating of MAFLD patients exhibited correlations with clinical indicators, akin to the positive association between *Rhodococcus* and BMI values reported in a previous study (34). Although our findings reveal disturbances in the tongue coating microbiota of MAFLD patients in both animal and clinical studies, along with the identification of certain highly abundant microbial taxa, the specific relationship between microbiota and MAFLD necessitates further extensive investigation for exploration and validation. We believe that as related research advances and emerging technologies develop, the connections between MAFLD and tongue coating microecology will become clearer, ultimately providing novel insights for the prevention and treatment of MAFLD.

### Discussion on the effect of WMT treatment on the microbiota of MAFLD tongue coating

In this study, we found that after WMT treatment, the abundance of *Peptostreptococcus* in the tongue coating of MAFLD patients significantly decreased, while the abundance of *Lachnospiraceae* and *Bifidobacterium* increased markedly. A study demonstrated that FMT effectively alleviates symptoms in patients with diarrhea-predominant irritable bowel syndrome, leading to a significant increase in *Bifidobacterium* abundance in the intestinal microbiota (35). This finding aligns with our observation of a substantial increase in *Bifidobacterium* in MAFLD patients following WMT. Furthermore, Wu (36) reported that *Bifidobacterium* is negatively correlated with metabolic endotoxemia, suggesting that this genus may help mitigate disease progression. While *Peptostreptococcus* was typically considered part of the normal human microbiota, it has also been associated with infections in various tissues and organs. Therefore, based on these findings, we infer that WMT not only impacts gut microbiota but also influences the microbiota present in the tongue coating.

In addition, this study unexpectedly identified a correlation between tongue coating microorganisms and the efficacy of WMT treatment for MAFLD. For instance, *Porphyromonas* was found to be positively correlated with obesity, ALT, AST, and disease severity in MAFLD patients, consistent with findings reported in other studies. Specifically, *Porphyromonas gingivalis* has been closely associated with the onset and progression of various diseases, including MAFLD (37, 38), cirrhosis (39), and liver cancer (40). This relationship may stem from the ability of *Porphyromonas* to invade the intestine, leading to an imbalance in gut microbiota and increased serum endotoxin levels. Consequently, this disruption may compromise the intestinal mucosal barrier and interfere with hepatic fat metabolism (41). These findings suggest that *Porphyromonas*, *Peptostreptococcus*, and *Bifidobacterium* may serve as potential biomarkers for predicting the efficacy of WMT in treating MAFLD. To further investigate this, we conducted animal experiments that yielded similar results. Although no significant differences were observed in the microbiota of the tongue coating between mice receiving healthy bacterial solutions and those receiving severe fatty liver bacterial solutions after FMT, *Enterococcus* exhibited a higher abundance in the group receiving the severe fatty liver bacterial solution. As a well-known conditional pathogen, *Enterococcus* is recognized as a leading cause of infection (42). A clinical study conducted by Vieira (43) demonstrated that intestinal *Enterococcus* can translocate to the liver and other tissues, while Schwenger KJP (44) found a strong correlation between *Enterococcus* abundance and disease severity and mortality in MAFLD patients. Based on these findings, we speculate that *Enterococcus* may be widely present in MAFLD in both animal models and humans, potentially acting as a conditional pathogen closely linked to the onset and progression of MAFLD.

However, this study has several limitations, including a small sample size and microbiological assessments of tongue coating at different treatment cycles. Therefore, there is an urgent need for multicenter, large-scale studies to validate the potential significance of these microorganisms in MAFLD. Furthermore, the metabolic products of tongue coating microorganisms are relatively abundant, and MAFLD patients exhibit their own accumulation of metabolic products, some of which were closely related to specific microorganisms.

To address these complexities, integrating and analyzing the metabolomics of both tongue coating microbiota and intestinal microbiota could lead to the identification of additional and more robust potential biomarkers. Such an approach may provide new diagnostic methods for MAFLD, offer important theoretical insights into the mechanisms underlying MAFLD, and pave the way for innovative strategies in the prevention and treatment of this condition.

In addition, this study acknowledges several limitations. Firstly, the sample size was relatively small, which may limit the generalizability of the findings. Secondly, despite the observed associations, there remains a dearth of specific mechanistic research elucidating the relationship between tongue coating microorganisms and MAFLD. Consequently, there is a pressing need for multi-center studies with larger sample sizes to comprehensively investigate the effects of WMT, on the tongue coating microbiota and the clinical efficacy in MAFLD patients. Furthermore, future research should endeavor to explore the potential microbial interplay between the tongue coating and the gut, as well as delve deeper into the underlying mechanisms to enhance our understanding of this intricate relationship.dd.

## Conclusion

In summary, WMT demonstrates a substantial capacity to ameliorate the hepatic steatosis index among patients with MAFLD, accompanied by a minimal occurrence of adverse reactions, thereby suggesting its safety and efficacy as a therapeutic intervention for MAFLD. Notably, significant disparities were observed in the tongue coating microbiota of both MAFLD patients and mice, in contrast to healthy controls. Various bacterial genera, including *Streptococcus*, *Porphyromonas*, *Johannes*, and *Rhodococcus*, exhibited close correlations with clinical indicators of MAFLD in the tongue coating of affected patients, hinting at their potential as biological markers for assessing disease severity. Furthermore, WMT exerts a notable influence on the tongue coating microbiota of MAFLD patients and mice. After WMT, multiple bacterial genera, such as *Bifidobacterium*, *Streptococcus*, and *Porphyromonas*, were found to be associated with clinical indicators of MAFLD, indicating their utility as biological markers for evaluating the therapeutic efficacy of WMT in treating MAFLD.

## Data Availability

The original contributions presented in the study are included in the article/supplementary material. Further inquiries can be directed to the corresponding author/s.
